# Optimal timing of post-therapeutic [^131^I]NaI whole-body scintigraphy in predominantly low- and intermediate-risk differentiated thyroid cancer: a secondary analysis of prospectively acquired data and a structured literature review

**DOI:** 10.1186/s12880-026-02647-y

**Published:** 2026-08-08

**Authors:** Andreas Pfestroff, Hannah Wolfram, Kathrin Pfestroff, Wadim Bowl, Ali Ebrahimifard, Shamim Bagheri, Pietro di Fazio, Behrooz H. Yousefi, Elisabeth Maurer, Katharina Holzer, Glenn Flux, Jan Taprogge, Markus Luster, Friederike Eilsberger

**Affiliations:** 1 Department of Nuclear Medicine, Marburg University, University Hospital Marburg, Marburg, Germany; 2 Department of Visceral, Thoracic and Vascular Surgery, Marburg University, University Hospital Marburg, Philipps University Marburg, Marburg, Germany; 3 Joint Department of Physics, The Royal Marsden NHS Foundation Trust, The Institute of Cancer Research, Sutton, UK; 4 The Institute of Cancer Research, London, UK

**Keywords:** Differentiated thyroid cancer, Radioiodine therapy, Post-therapeutic whole-body scintigraphy, Acquisition timing, Image quality, Reader agreement

## Abstract

**Background:**

Post-therapeutic [^131^I]NaI whole-body scintigraphy (ptWBS) is mandatory after radioiodine therapy (RAIT), but the most suitable acquisition time remains uncertain. We compared image interpretability at early and delayed time points in patients with differentiated thyroid cancer (DTC).

**Methods:**

This retrospective secondary analysis included prospectively acquired ptWBS data from 35 patients. Scans obtained at 6, 24, 48, 72, and 168 h after RAIT were independently ranked by three nuclear medicine physicians and evaluated with a 10-item criteria-based image-quality score. Rankings were analyzed using a Plackett-Luce model, and criteria-based scores were analyzed using generalized estimating equations.

**Results:**

The analysis comprised 167 scans, 501 criteria-based ratings, and 500 intuitive rank observations. The 24-h acquisition had the highest estimated preference, followed by 48, 6, 72, and 168 h. After Holm adjustment, 24 h was favored over 6, 72, and 168 h, whereas the difference between 24 and 48 h was not significant (*P* = 0.091). The criteria-based score was highest at 24 h (39.52; 95% CI, 38.62–40.43) and next-highest at 48 h (34.79); all pairwise differences in scores were significant. Each 5-point increase in the criteria-based score was associated with higher odds of a more favorable intuitive ranking (OR, 2.01; 95% CI, 1.70–2.38).

**Conclusions:**

PtWBS acquired at 24 h provided the best overall interpretability of the images. A 48 h scan may be considered when practical or radiation-protection considerations favor later imaging, but equivalence to 24 h has not been established. Additional delayed imaging may still be useful when distant metastatic disease is suspected.

## Introduction

Although the indication for adjuvant radioiodine therapy (RAIT) in patients with low- and intermediate-risk differentiated thyroid cancer (DTC) has recently been debated, RAIT remains one of the most frequently performed nuclear medicine therapies and an established component of DTC management [1]. Post-therapeutic [^131^I]NaI whole-body scintigraphy (ptWBS) is considered as mandatory by national and international guidelines to document the distribution of administered iodine [2, 3]. The findings may contribute to staging, dynamic risk stratification, estimation of recurrence risk, and adaptation of subsequent follow-up [4–7]. In particular, the detection of metastatic disease may affect both prognosis and the need for further therapeutic interventions.

Serum thyroglobulin alone does not reliably exclude iodine-avid disease. Campennì et al. showed that a stimulated thyroglobulin concentration below 1 ng/ml did not rule out metastases [8], and Thai et al. reported only a weak association between postoperative serum thyroglobulin and ptWBS findings [9]. Souza Rosário et al. found that ptWBS resulted in revision of the disease stage in 8.3% of patients and prompted a change in treatment strategy in a further 15% [10]. Low-activity pretherapeutic diagnostic whole-body scans may also have lower sensitivity, particularly for lymph-node and pulmonary metastases. Iwano et al. reported concordance rates between [^123^I]NaI diagnostic WBS and [^131^I]NaI ptWBS of 89% for thyroid-bed involvement and 86% for bone metastases, but only 61% for lymph-node and 39% for lung metastases [11]. Similar differences have been described in other studies [12, 13], and ptWBS has also been shown to contribute to early risk assessment and prognostication [14].

Despite approximately 80 years of clinical experience with RAIT, the optimal timing of ptWBS remains uncertain [15, 16]. National and international guidelines provide heterogeneous recommendations: the American Thyroid Association (ATA), European Thyroid Association (ETA), British Thyroid Association (BTA), Latin American Thyroid Society (LATS), and Society of Nuclear Medicine and Molecular Imaging (SNMMI)/ European Association of Nuclear Medicine (EANM) describe time windows ranging from approximately 2 to 12 days, whereas the German and Japanese guidelines do not specify a particular acquisition time [2, 3, 15, 17–21]. Published studies have likewise used different time points [10, 12, 13, 22]. Most investigations specifically addressing timing compared scans acquired on day 3 or later [23–28]; only one adult study beginning on day 2 and one pediatric study included earlier serial imaging [29, 30].

The primary aim of the present study was therefore to compare the overall interpretability of ptWBS acquired between 6 and 168 h after RAIT. Images were assessed first by an intuitive ranking and subsequently using predefined criteria and a structured image-quality score. Secondary objectives were to examine the association and interreader agreement between the two assessment approaches and to evaluate the visibility of iodine-avid remnants and metastatic lesions descriptively. A structured review of the available literature was performed to place the findings in context.

## Materials and methods

### Study design and patients

This study was a retrospective secondary analysis of prospectively acquired imaging data from the Marburg cohort of the multinational MEDIRAD project [31]. Adult patients with histologically confirmed DTC who underwent their first RAIT at our tertiary referral center between June 2019 and August 2020 were eligible. All patients had undergone either near-total thyroidectomy or completion thyroidectomy after initial hemithyroidectomy.

Exclusion criteria for the parent imaging protocol included previous therapeutic radiation exposure; recent administration of radionuclides, medications, or iodinated contrast media expected to interfere with iodine metabolism within the preceding three months; a recent diagnostic radioiodine scan; previous external-beam radiotherapy; or systemic chemotherapy. All patients were considered to have low- or intermediate-risk disease before RAIT. In one patient, iodine-avid bone metastases were first identified on post-therapeutic imaging, resulting in classification as M1.

### Radioiodine therapy and imaging protocol

A median activity of 3.7 GBq (range, 2.5–3.8 GBq) of [^131^I]NaI was administered after thyroid-stimulating hormone (TSH) stimulation. Stimulation was achieved with recombinant human thyrotropin in all but one patient, in whom thyroid hormone withdrawal had been initiated before referral. Routine laboratory measurements included TSH, postoperative thyroglobulin, stimulated thyroglobulin, and anti-thyroglobulin antibodies.

Whole-body scans were scheduled at 6 h (± 2 h), 24 h (± 4 h), 48 h (± 4 h), 72 h (± 12 h), optionally 96 h (± 12 h), and 168 h (± 24 h) after administration. Images were acquired on a Siemens Symbia S gamma camera using high-energy general-purpose parallel-hole collimators. A 364-keV photopeak with a symmetric 20% energy window was used. Identical acquisition settings were used at all time points. The whole body planar imaging (anterior/posterior views) were performed using a zoom factor of 1, a 1024 × 256 matrix and a scanning speed of 20 cm/min. The 96 h acquisition was excluded from comparative analyses because only four scans were available.

### Image assessment

Three board-certified nuclear medicine physicians independently reviewed the ptWBS examinations. The intuitive and criteria-based reading sessions were separated by several months. The intuitive ranking was completed first so that the initial overall judgment was not influenced by prior use of the structured criteria. Readers were blinded to each other’s assessments.

For the intuitive assessment, each reader ranked the available scans for an individual patient from best to worst based on overall image interpretability. Rank 1 denoted the most favorable image. Because the number of available time points varied across patients, these assessments reflected partial within-patient rankings rather than independent numerical scores.

For the criteria-based assessment, each reader rated ten predefined components on a five-point Likert scale: visualization of the thyroid bed, cervical lymph-node regions, mediastinum, lungs, bones, liver, counts per pixel, target-to-background ratio, whole-body contour, and blood-pool activity. Each item was scored from 1 (insufficient) to 5 (very good), resulting in a total score of 10–50 points, with higher values indicating better image quality. The criteria were based on principles described by Van Nostrand and Atkins [32]. The physicians scored the visualization quality of the bone and lung area on basis of how likely radioiodione-avid lesions would have had chance of becoming detectable if they were present. In general, this is most likely when bone and lung background activity is low but still detectable as the skeletal or lung region (shape/contour). 

The absence of distant metastases in all these M0 patients is still concordant to the most recent follow-up data.

### Exploratory lesion-visibility analysis

For the exploratory lesion-based analysis, the serial scans were reviewed for iodine-avid thyroid remnant foci, lymph node metastases, and bone metastases. Foci were matched across time points by anatomical location, and all corresponding foci seen on at least one scan were combined into a composite within-patient reference set. At each acquisition time, every focus in this set was recorded as visible or not visible. The denominator at each time point therefore included only lesions from patients with a scan available at that time.

Because multiple lesions originated from the same patient and only one patient had bone metastases, lesion-visibility results were considered descriptive. No formal claim of diagnostic sensitivity or specificity was made, and the composite reference set was not regarded as an independent gold standard.

### Statistical analysis

The intuitive rankings and criteria-based scores were analyzed separately. Rankings were evaluated with a Plackett-Luce model, which estimates the relative probability that a scan acquired at one time point is ranked ahead of scans acquired at the other available time points. Results are reported as normalized worth estimates and pairwise preference odds ratios. Because each patient contributed assessments from three readers, standard errors and confidence intervals were calculated with patient-level clustering. Pairwise comparisons were performed after an overall test of acquisition time, and P values were adjusted with the Holm procedure.

Criteria-based scores were treated as numerical outcomes and compared with a Gaussian generalized estimating equation model, using patient as the clustering variable. Acquisition time, reader, and the acquisition-time-by-reader interaction were included in the model. Reader-averaged estimated means and pairwise mean differences are reported with 95% confidence intervals. Pairwise P values were adjusted with the Holm procedure.

The relationship between the two assessment approaches was examined with rank-ordered logistic regression. The effect is reported as an odds ratio per 5-point increase in the criteria-based score; a second model additionally adjusted for acquisition time. Interreader reliability of the criteria-based score was assessed with absolute-agreement intraclass correlation coefficients for a single reader, ICC(A,1), and for the mean of three readers, ICC(A,3). Confidence intervals were obtained from 1,000 patient-level bootstrap samples. Agreement among the intuitive rankings was summarized by the mean patient-specific Kendall’s W and by the proportion agreeing on the top-ranked acquisition time.

Missing observations were not imputed. All available assessments were included in the respective analyses. Statistical tests were two-sided, and *P* < 0.05 was considered statistically significant. Confidence intervals for pairwise comparisons were not multiplicity-adjusted, whereas the corresponding P values were adjusted using the Holm method. Analyses were performed using Python 3.13.5 with NumPy 2.3.5, SciPy 1.17.0, statsmodels 0.14.6, Excel 16.99.1, and SigmaPlot 15.0.

### Structured literature review

Two complementary PubMed searches were performed using terms for DTC, radioiodine, post-therapeutic whole-body scintigraphy, and acquisition timing. The search was last updated on 14.07.2026. Studies comparing at least two post-therapeutic imaging time points were eligible. Adult and pediatric studies were summarized separately. After overlap between the two searches had been removed, eight unique studies remained. Because the studies differed substantially in patient populations, imaging protocols, and outcome definitions, the findings were summarized descriptively; no meta-analysis or formal risk-of-bias assessment was performed.

Search string 1: ((“Thyroid Neoplasms” OR “differentiated thyroid carcinoma” OR “differentiated thyroid cancer”) AND (“Iodine Radioisotopes” OR I-131 OR 131I OR iodine-131 OR radioiodine) AND (“whole body scan” OR “whole-body scan” OR “whole body scintigraphy” OR scintigraphy OR scan) AND (posttherapy OR post-therapy OR posttherapeutic OR post-therapeutic) AND (day OR delayed OR early OR timing OR sequential OR comparison)).

Search string 2: ((“Thyroid Neoplasms” OR differentiated thyroid cancer OR differentiated thyroid carcinoma OR DTC) AND (“Iodine Radioisotopes” OR I-131 OR iodine-131 OR radioiodine) AND ((post-therapy OR posttherapeutic OR post-treatment) AND (“whole body scan” OR whole-body scintigraphy OR WBS) AND (early OR delayed OR timing OR optimal OR sequential OR comparison))).

## Results

### Patient characteristics and available imaging data

Thirty-five patients were included. The median age was 47 years (range, 20–76 years), 25 patients (71%) were female, and 31 (89%) had papillary thyroid carcinoma. Baseline characteristics are summarized in Table 1. Even though one patient showed bone metastases on imaging, all patients were classified as low- or intermediate-risk according to the ATA prior to radioiodine therapy.

At 6, 24, 48, 72, 96, and 168 h, scans were available for 35, 35, 35, 30, 4, and 32 patients, respectively (Table 2). After exclusion of the 96 h time point, 167 scans remained for analysis. These generated 501 criteria-based score ratings and 500 intuitive rank observations. All available scans had complete criteria-based ratings from all three readers. One intuitive rank was missing for the 168-h scan of one patient and one reader and was omitted only from analyses involving intuitive ranking.

**Table 1:** Baseline characteristics and availability of post-therapeutic whole-body scans

Characteristic	Value
Sex	Female: 25 (71%); male: 10 (29%)
Age, years	Median 47 (range, 20–76)
Histology	Papillary: 31 (89%); follicular: 4 (11%)
T category	T1: 23 (66%); 1a:9 1b:14 T2: 8 (23%) T3: 4 (11%); 3a: 1 3b: 2 not specified: 1
N category	Nx: 17 (49%); N0: 7 (20%); N1a: 8 (23%); N1b: 3 (9%)
M category	M0: 34 (97%); M1: 1 (3%)
Available scans	6 h: 35 (100%); 24 h: 35 (100%); 48 h: 35 (100%); 72 h: 30 (86%); 96 h: 4 (11%); 168 h: 32 (91%)
Administered [^131^I]NaI activity	Median 3.7 GBq (range, 2.5–3.8)
Stimulated TSH, mU/L	Median 125 (range, 62–259)
Postoperative unstimulated Tg, ng/mL*	Median 0.35 (range, 0-4.9)
Stimulated Tg, ng/mL*	Median 7.05 (range, 0–92)

*Patients with anti-thyroglobulin antibodies were excluded from thyroglobulin summaries. Tg, thyroglobulin; TSH, thyroid-stimulating hormone

**Table 2:** Descriptive image-assessment results by acquisition time point. Rank 1 indicates the best image, whereas higher criteria-based scores indicate better image quality. Percentages for “ranked first” are based on the available intuitive ratings at each time point

Time	Available scans	Criteria score, mean ± SD	Median [IQR]	Mean intuitive rank	Ranked first, *n* (%)
6 h	35	31.50 ± 6.30	32.0 [28.0–36.0]	2.76	14 (13.3%)
24 h	35	39.52 ± 5.79	39.0 [36.0–43.0]	1.74	52 (49.5%)
48 h	35	34.74 ± 7.01	35.0 [30.3–40.0]	2.25	33 (32.4%)
72 h	30	26.91 ± 6.82	27.0 [22.0–31.0]	3.46	5 (5.4%)
168 h	32	14.43 ± 3.95	13.0 [12.0–16.0]	4.47	1 (1.1%)

### Descriptive assessment results

**Fig. 1:**
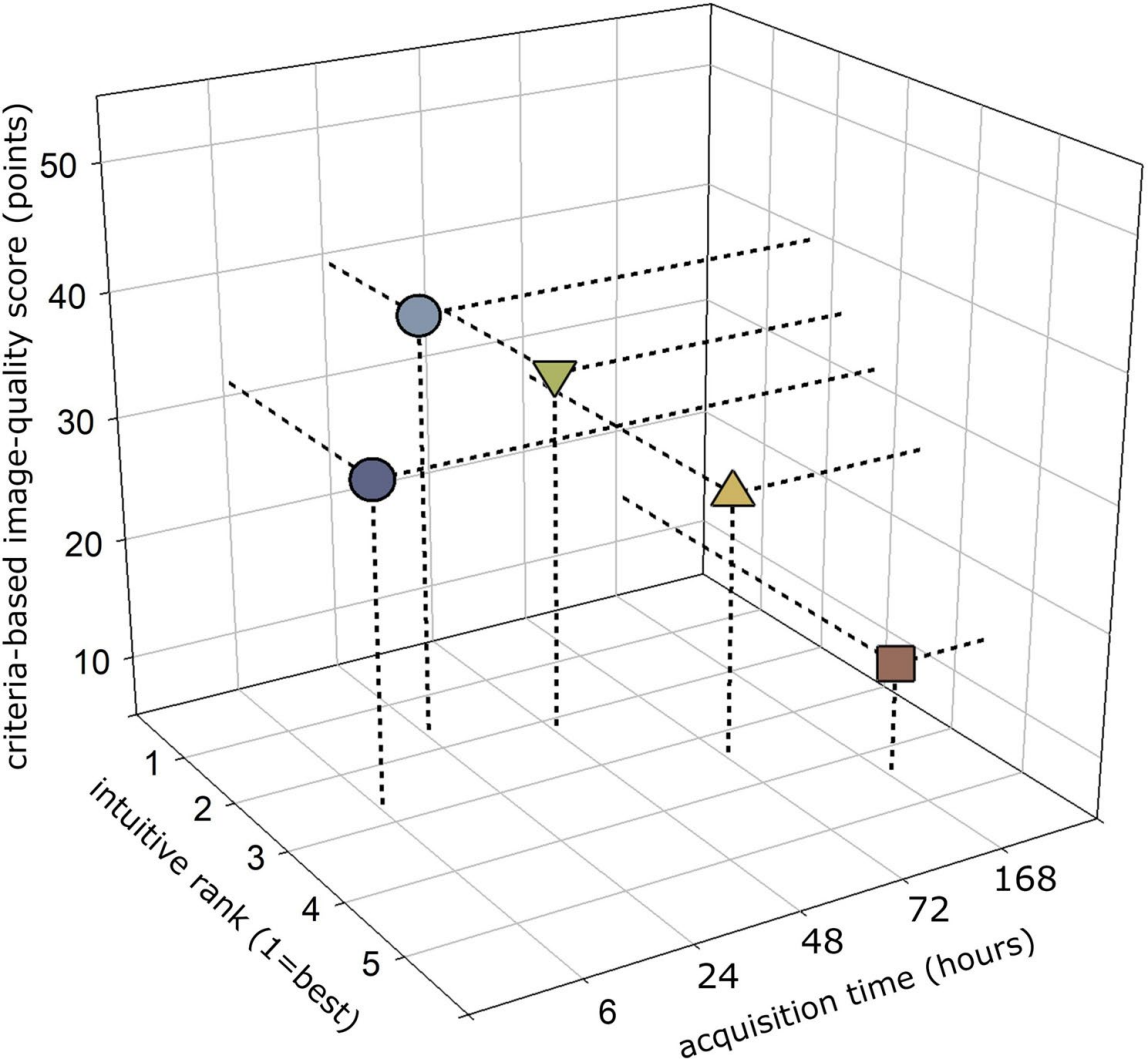
Descriptive relationship between acquisition time, mean intuitive rank, and mean criteria-based image-quality score. Each point represents the reader-averaged value at one acquisition time

Both assessment approaches identified 24 h as the most favorable acquisition time and 168 h as the least favorable. The overall reader-averaged order was 24 h, 48 h, 6 h, 72 h, and 168 h. Figure 1 provides a descriptive overview of this pattern.

### Intuitive ranking

Acquisition time had a significant overall effect on intuitive ranking (cluster-robust Wald χ²(4) = 73.48; *P* < 0.001). The normalized worth was highest at 24 h, followed by 48 h, 6 h, 72 h, and 168 h (Table 3).

**Table 3:** Plackett-Luce model estimates for intuitive image ranking. Higher normalized worth indicates a greater model-estimated probability of being ranked ahead of other available acquisition times. Preference odds are shown relative to the 168-h acquisition

Acquisition time	Normalized worth (95% CI)	Preference odds vs. 168 h (95% CI)
6 h	0.161 (0.118–0.208)	8.75 (4.51–16.97)
24 h	0.487 (0.364–0.597)	26.43 (12.01–58.14)
48 h	0.249 (0.154–0.369)	13.50 (5.86–31.13)
72 h	0.085 (0.053–0.127)	4.62 (2.46–8.68)
168 h	0.018 (0.009–0.036)	Reference

When all five acquisition times were available, the estimated probability of being ranked first was 48.7% for 24 h and 1.8% for 168 h. The magnitude of time-point preferences differed among readers (χ²(8) = 22.82; *P* = 0.0036), although all three readers assigned the highest worth to 24 h and the lowest worth to 168 h.

**Table 4:** Pairwise comparisons of acquisition times based on intuitive ranking. An odds ratio greater than 1 indicates that the first-listed time was more likely to be ranked ahead of the second-listed time. Confidence intervals are unadjusted; P values were adjusted for ten pairwise comparisons using the Holm method

Pairwise comparison	Preference odds ratio (95% CI)	Holm-adjusted *P*
6 h vs. 24 h	0.33 (0.24–0.47)	< 0.001
6 h vs. 48 h	0.65 (0.34–1.23)	0.183
6 h vs. 72 h	1.89 (1.09–3.29)	0.070
6 h vs. 168 h	8.75 (4.51–16.97)	< 0.001
24 h vs. 48 h	1.96 (1.01–3.78)	0.091
24 h vs. 72 h	5.72 (3.00-10.91)	< 0.001
24 h vs. 168 h	26.43 (12.01–58.14)	< 0.001
48 h vs. 72 h	2.92 (1.92–4.46)	< 0.001
48 h vs. 168 h	13.50 (5.86–31.13)	< 0.001
72 h vs. 168 h	4.62 (2.46–8.68)	< 0.001

After multiplicity adjustment, 24 h was significantly favored over 6, 72, and 168 h (Table 4). The intuitive ranking did not provide sufficient evidence to distinguish 24 h from 48 h (Holm-adjusted *P* = 0.091).

### Criteria-based image-quality score

The reader-averaged criteria-based score differed significantly across acquisition times (χ²(4) = 1753.29; *P* < 0.001). Model-estimated means are shown in Table 5.

**Table 5:** Reader-averaged criteria-based image-quality scores by acquisition time point. Estimates were obtained from a generalized estimating equation model accounting for repeated assessments within patients and were averaged equally across the three readers

Acquisition time	Reader-averaged adjusted mean (95% CI)
6 h	31.50 (30.01–32.98)
24 h	39.52 (38.62–40.43)
48 h	34.79 (33.12–36.47)
72 h	26.86 (24.99–28.73)
168 h	14.37 (13.32–15.42)

**Table 6:** Pairwise differences in criteria-based image-quality scores. Positive values indicate a higher mean score for the first-listed time; negative values indicate a lower mean score. Confidence intervals are unadjusted; P values were adjusted using the Holm method

Pairwise contrast	Adjusted mean difference (95% CI)	Holm-adjusted *P*
6–24 h	-8.03 (-9.47 to -6.58)	< 0.001
6–48 h	-3.30 (-6.04 to -0.56)	0.018
6–72 h	4.64 (2.05 to 7.22)	< 0.001
6–168 h	17.12 (15.58 to 18.67)	< 0.001
24–48 h	4.73 (2.93 to 6.53)	< 0.001
24–72 h	12.66 (10.74 to 14.58)	< 0.001
24–168 h	25.15 (23.93 to 26.38)	< 0.001
48–72 h	7.93 (6.52 to 9.34)	< 0.001
48–168 h	20.42 (18.50 to 22.34)	< 0.001
72–168 h	12.49 (10.52 to 14.46)	< 0.001

All criteria-score comparisons remained statistically significant after Holm correction (Table 6). A significant acquisition-time-by-reader interaction was present (χ²(8) = 107.62; *P* < 0.001), indicating that the magnitude of score differences varied among readers. Nevertheless, each reader assigned the highest mean score to 24 h and the lowest mean score to 168 h. The relative ordering of 6 and 48 h differed between reader 1 and readers 2 and 3.

### Association between assessment approaches

**Fig. 2:**
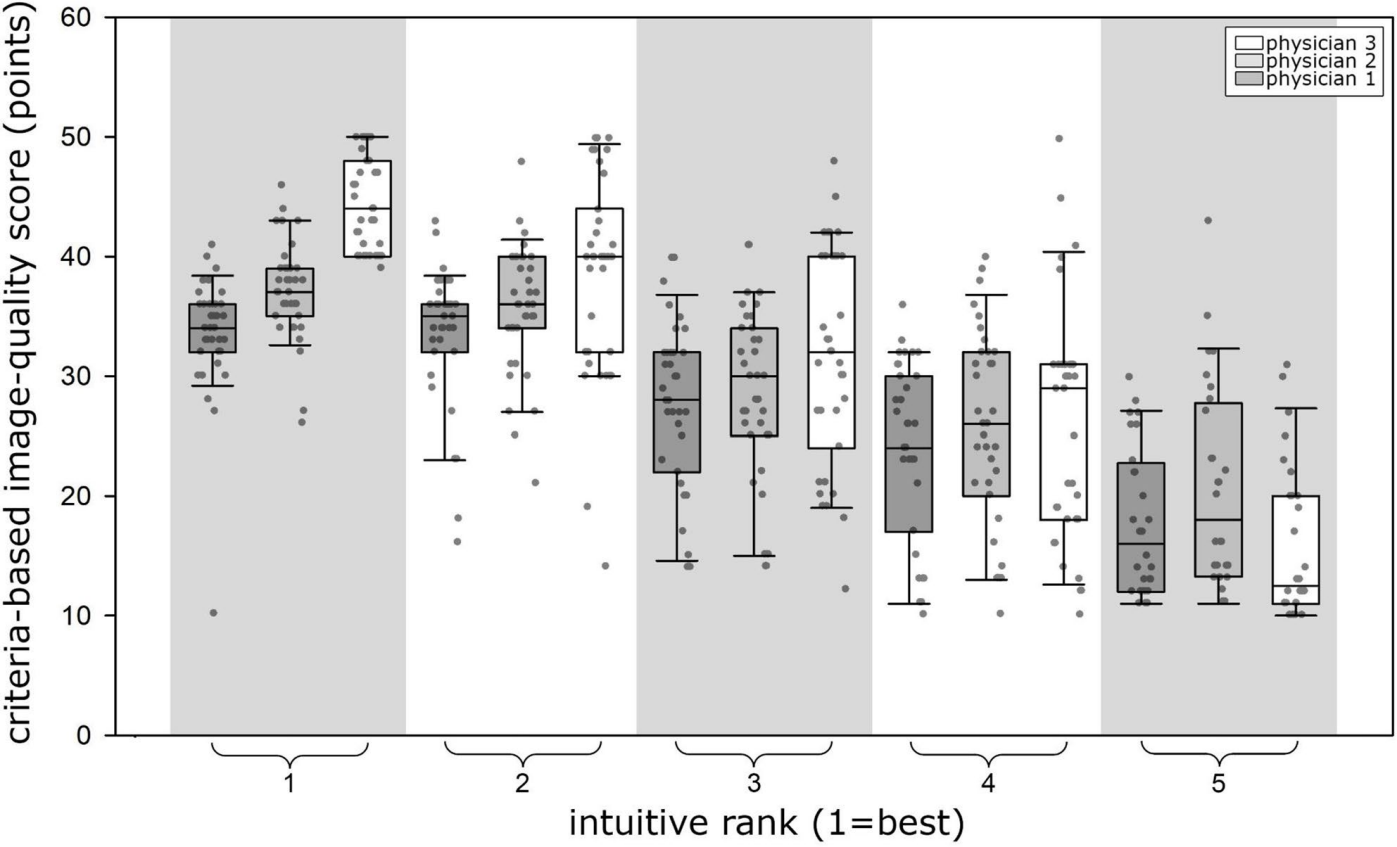
Relationship between intuitive ranking and the criteria-based image-quality score. Box plots show score distributions at each intuitive rank for the three readers, with individual observations overlaid. Rank 1 denotes the most favorable image; boxes show the interquartile range and the central line the median

Higher criteria-based scores were strongly associated with more favorable intuitive rankings. Each 5-point increase in the criteria-based score approximately doubled the odds that an image would be ranked ahead of another available image (OR, 2.01; 95% CI, 1.70–2.38; *P* < 0.001). The association remained significant after adjustment for acquisition time (adjusted OR, 1.72; 95% CI, 1.36–2.18; *P* < 0.001), indicating that it was not explained solely by the common effect of acquisition time (Fig. 2).

### Interreader agreement

Across all images, absolute agreement for the criteria-based score was ICC(A,1) = 0.733 (95% CI, 0.683–0.775) for a single reader and ICC(A,3) = 0.892 (95% CI, 0.866–0.912) for the mean of three readers. Time-specific ICCs were lower (Table 7), showing that the large differences between acquisition times contributed to the high overall agreement. The very low ICC at 24 h likely reflects the narrow range of scores at this generally favorable time point.

**Table 7:** Interreader reliability of the criteria-based image-quality score. Absolute-agreement intraclass correlation coefficients are reported for a single reader, ICC(A,1), and for the mean of three readers, ICC(A,3). Confidence intervals were obtained by patient-level bootstrap resampling

Acquisition time	ICC(A,1), single reader (95% CI)	ICC(A,3), mean of three readers (95% CI)
Overall	0.733 (0.683–0.775)	0.892 (0.866–0.912)
6 h	0.234 (0.032–0.433)	0.479 (0.091–0.696)
24 h	0.009 (-0.052-0.092)	0.028 (-0.174-0.233)
48 h	0.315 (0.169–0.447)	0.580 (0.379–0.708)
72 h	0.419 (0.193–0.577)	0.684 (0.418–0.804)
168 h	0.352 (0.152–0.504)	0.620 (0.349–0.753)

For the intuitive rankings, the mean patient-specific Kendall’s W was 0.823 (95% CI, 0.778–0.864). All three readers selected the same top-ranked acquisition time in 37.1% of patients, and at least two of the three readers agreed on the top-ranked time in 91.4%.

### Exploratory lesion visibility

The proportions of composite-reference lesions visible at each acquisition time are summarized in Table 8. Thyroid-remnant foci and lymph-node lesions were most frequently visible between 24 and 72 h. Visibility was lower at 6 and 168 h. In the single patient with bone metastases, all six lesions were visible at 24 h; fewer were visible at the other time points. Because lesions were clustered within patients and metastatic lesion counts were very small, these findings are descriptive and should not be interpreted as estimates of diagnostic sensitivity.

**Table 8:** Exploratory visibility of iodine-avid foci at each acquisition time. Values are shown as visible lesions/composite-reference lesions available for evaluation at that time, percentage, and exact binomial 95% confidence interval. The composite reference comprised anatomically corresponding foci seen on at least one serial post-therapeutic scan

Lesion category	6 h	24 h	48 h	72 h	168 h
Thyroid-remnant foci	48/70 (68.6%; 95% CI, 56.4–79.1)	66/70 (94.3%; 95% CI, 86.0-98.4)	69/70 (98.6%; 95% CI, 92.3–100.0)	63/63 (100.0%; 95% CI, 94.3–100.0)	52/65 (80.0%; 95% CI, 68.2–88.9)
Lymph-node metastases	0/4 (0.0%; 95% CI, 0.0-60.2)	3/4 (75.0%; 95% CI, 19.4–99.4)	4/4 (100.0%; 95% CI, 39.8–100.0)	3/3 (100.0%; 95% CI, 29.2–100.0)	2/4 (50.0%; 95% CI, 6.8–93.2)
Bone metastases*	4/6 (66.7%; 95% CI, 22.3–95.7)	6/6 (100.0%; 95% CI, 54.1–100.0)	4/6 (66.7%; 95% CI, 22.3–95.7)	4/6 (66.7%; 95% CI, 22.3–95.7)	3/6 (50.0%; 95% CI, 11.8–88.2)

*All bone lesions originated from one patient; no inferential analysis was performed

### Structured literature review

**Fig. 3:**
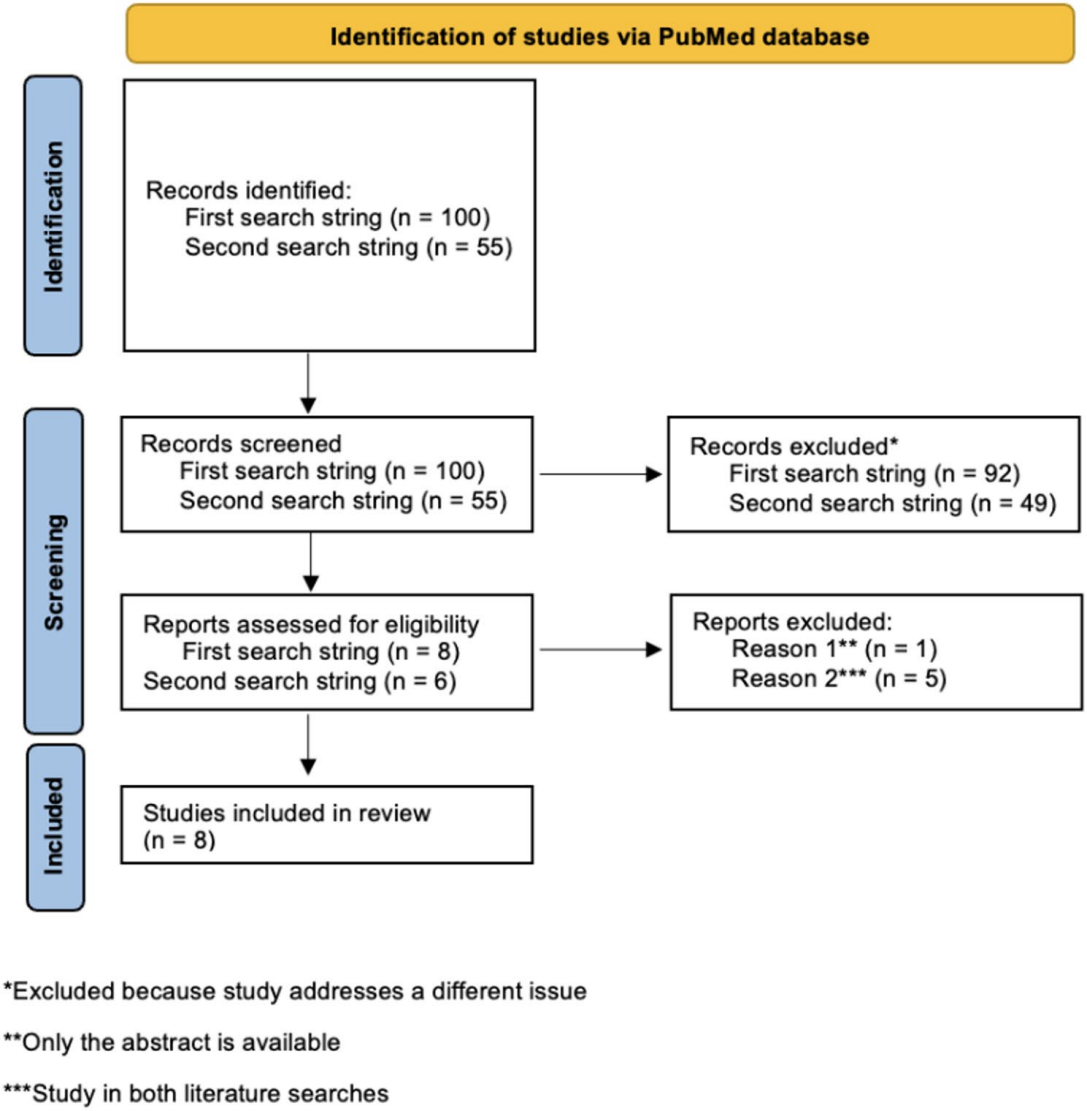
Flow diagram of the structured PubMed search. One study was identified by both search strategies; eight unique previous studies met the inclusion criteria

The two PubMed searches identified 100 and 55 records, respectively. After screening, eligibility assessment, and removal of overlap, eight unique previous studies were included: seven adult studies and one pediatric study (Fig. 3; Table 9). The studies differed in risk profile, administered activity, acquisition protocol, and outcome definition. Earlier imaging tended to favor visualization of thyroid remnants, whereas several studies reported additional value of delayed imaging for pulmonary or osseous metastases.

**Table 9:** Previous studies evaluating the timing of post-therapeutic [^131^I]NaI whole-body scintigraphy

Study	Population, risk profile, and design	Acquisition times	Reference endpoint and main conclusion
Hung et al., 2009 [25]	239 adults; mixed risk; retrospective	Days 3–4, 5–6, and 10–11	Lesion visibility: Lesion visualization decreased on late sequential images; late images missed subsets of lymph-node, lung, and bone lesions.
Chong et al., 2010 [23]	52 adults; high risk/M1; retrospective	Days 3 and 7	Additional lesion detection: Day-7 imaging identified more lung and bone metastases.
Lee et al., 2011 [24]	81 adults; mixed risk; retrospective	Days 3 and 10	Uptake ratio and lesion detectability: Iodine-avid lesions generally showed higher uptake ratios and were more readily detected on early images.
Kodani et al., 2012 [28]	24 adults; intermediate/high risk; retrospective	Days 3 and 7–9	Additional lesion detection: Delayed imaging increased detection of lung and bone metastases.
Salvatori et al., 2013 [27]	134 adults; mixed risk; prospective	Days 3 and 7	Discordant lesion visibility between time points: Using only a single time point could miss thyroid remnants, lymph node metastases, or distant metastases in approximately 20% of patients.
Qiu et al., 2020 [29]	137 adults; intermediate/high risk; retrospective	Days 2, 3, and 4	Additional metastatic lesion detection: Day-4 imaging detected additional lymph-node, pulmonary, and bone metastases.
Liu et al., 2022 [26]	161 adults; intermediate/high risk; retrospective	Days 3 and 7/10	Remnant and lung-lesion visibility: Day-3 imaging favored thyroid-remnant visualization, whereas later imaging favored lung-metastasis detection.
Kumar et al., 2024 [30]	95 pediatric/young adult patients; mixed risk; retrospective	Days 1, 2, and 3	Overall image quality and lesion detectability: Day-3 post-therapeutic imaging was considered optimal.
Present study	35 adults; predominantly low/intermediate risk; secondary analysis of prospective data	6, 24, 48, 72, and 168 h	Overall interpretability and criteria-based image quality: Overall interpretability and criteria-based image quality were highest at 24 h; 48 h ranked second, and 168 h performed worst.

## Discussion

To our knowledge, no previous study has combined very early imaging at 6 and 24 h with serial acquisitions through day 7 and two separate reader-based assessments. Both analyses showed a clear effect of acquisition time. The 24 h scan was rated best and the 168 h scan worst. In the intuitive ranking, 24 h was preferred to 6, 72, and 168 h, but not significantly to 48 h after adjustment for multiple comparisons. The criteria-based score, which allowed finer gradation, was highest at 24 h and differed significantly from every other time point; 48 h also scored higher than 6 h. The two analyses therefore agreed on the overall order of the scans, although not on every pairwise comparison.

The criteria-based score remained associated with the intuitive ranking after adjustment for acquisition time. This suggests that the structured score and the initial overall judgment reflected similar features of image interpretability. Agreement among readers was high for the rankings. For the numerical score, agreement was good when all time points were considered together but lower within individual time points. In practical terms, the readers agreed more closely on the broad time-related differences than on the exact score for a particular image. The reader-by-time interaction is consistent with this pattern: all readers selected 24 h as best and 168 h as worst, but they differed in the size of the score differences.

Previous timing studies in adult DTC patients generally began on day 3; the study by Qiu et al., which included imaging on day 2, was the main exception [23–29]. Their findings were not uniform. Hung et al. and Lee et al. reported poorer lesion visualization on very late scans around day 10 [24, 25]. By contrast, Chong et al. and Kodani et al. found additional pulmonary or osseous lesions on delayed scans around day 7 [23, 28]. Liu et al. found that day 3 favored remnant visualization, whereas later imaging favored lung metastases [26]. Qiu et al. detected additional metastatic lesions through day 4 [29], and Salvatori et al. showed that lesions visible at one time point may be missed at another [27]. Taken together, these studies suggest that the preferred timing may depend on the clinical question: remnant and regional disease may be best assessed earlier, whereas delayed images can add information in selected patients with distant metastases.

Most earlier studies included many intermediate- or high-risk patients and focused on distant disease. Our cohort was different: patients had no known distant metastases before RAIT. In the exploratory lesion analysis, the highest proportions of visible remnant and lymph-node foci occurred between 24 and 72 h, while visibility was lower at 6 and 168 h. The bone-metastasis findings came from a single patient and cannot be generalized. These data therefore do not show that early imaging alone is adequate when distant metastatic disease is suspected; additional delayed imaging may still be useful in that setting.

Because all but one patient in this cohort was prepared with recombinant human thyrotropin, the results may not be fully transferable to patients prepared by thyroid hormone withdrawal. Faster systemic clearance and lower background activity after rhTSH may contribute to favorable target-to-background contrast at early time points, whereas the optimal timing may differ under hypothyroid conditions [33].

Timing also has practical consequences. Discharge schedules vary between institutions and countries. A later scan may require some patients to return to the hospital, whereas keeping a patient in hospital solely for imaging can also be undesirable. Earlier scans are expected to expose staff to more radiation during positioning because more activity is retained, although staff dose was not measured in this study. Against this background, 48 h may be a reasonable practical choice when local logistics or radiation-protection considerations favor later imaging. This should not be interpreted as proof that 48 h is equivalent or non-inferior to 24 h.

The interpretation of late images also depends on the acquisition protocol. Reduced retained activity and biological clearance lower the number of detected counts at later time points. As acquisition settings were unchanged, count statistics may therefore have contributed to the poorer appearance of the 168 h images, in addition to changes in biodistribution. The present study cannot separate these effects and a count-based acquisition method should be considered for future studies.

The present comparison was restricted to planar whole-body scintigraphy. In current practice, SPECT/CT can improve anatomical localization and may help distinguish thyroid remnants from cervical lymph-node disease when planar findings are equivocal [14, 15]. Hybrid imaging may reduce ambiguity caused by anatomical overlap, but it cannot fully compensate for poor count statistics or an unfavorable target-to-background ratio. The optimal timing of post-therapeutic SPECT/CT was not evaluated in this study.

Several limitations deserve mention. The cohort was small, came from a single center, and included only one patient with distant metastases. The contribution of acquisition settings and count statistics to time-dependent image quality could not be quantified from the current analysis. Some scheduled scans were missing, and the 96 h time point could not be included in the comparative analyses. Three experienced readers provided the ratings from the same institution, which limits generalizability to other settings. Readers derived the criteria-based score, and it has not been validated as an independent measure of diagnostic performance. Lesion visibility was assessed descriptively against the combined serial scans rather than an external reference standard. The literature review was also descriptive and did not include formal risk-of-bias assessment. Even so, the repeated imaging protocol and the use of two complementary reading approaches allowed a detailed comparison of image interpretability from 6 h to 7 days after RAIT.

## Conclusion

In this predominantly low- and intermediate-risk cohort, ptWBS at 24 h had the best overall interpretability and the highest criteria-based image-quality score. The 48 h scan ranked second and may be considered when practical or radiation-protection factors favor later imaging, but the study was not designed to establish equivalence or non-inferiority. These findings mainly concern remnant and regional disease; additional delayed imaging remains reasonable when distant metastases are suspected.

## Data Availability

Data is available from the first author up on reasonable request.

## Abbreviations

ATA: American Thyroid Association
BTA: British Thyroid Association
DTC: Differentiated thyroid cancer
EANM: European Association of Nuclear Medicine
ETA: European Thyroid Association
HEGP: High-energy general-purpose
ICC: Intraclass correlation coefficient
LATS: Latin American Thyroid Society
ptWBS: Post-therapeutic [^131^I]NaI whole-body scintigraphy
RAIT: Radioiodine therapy
rhTSH: Recombinant human thyrotropin
SNMMI: Society of Nuclear Medicine and Molecular Imaging
Tg: Thyroglobulin
Tg-Ab: Anti-thyroglobulin antibodies
TSH: Thyroid-stimulating hormone
